# Building PRM in sub-Saharan Africa

**DOI:** 10.3389/fresc.2022.910841

**Published:** 2022-08-10

**Authors:** Abena Yeboaa Tannor, Mary Elizabeth S. Nelson, Hannah K. Steere, Benedict Okoe Quao, Andrew J. Haig

**Affiliations:** ^1^ Department of Health Promotion and Disability, School of Public Health, College of Health Sciences, Kwame Nkrumah University of Science and Technology, Kumasi, Ghana; ^2^ Department of Physical Medicine and Rehabilitation, Medical College of Wisconsin, Milwaukee, WI, United States; ^3^ Department of Physical Medicine and Rehabilitation, Harvard Medical School, Spaulding Rehabilitation Hospital, Boston, MA, United States; ^4^ Ankaful Leprosy & General Hospital, Ankaful, Ghana; ^5^ National Leprosy Control Programme, Disease Control & Preventive Department, Ghana Health Service Public Health Division, Korle-Bu, Accra, Ghana; ^6^ Department of Physical Medicine and Rehabilitation, University of Michigan, Ann Arbor, MI, United States

**Keywords:** physical and rehabilitation medicine, fellowship program, curriculum, training, Sub-Saharan Africa, Ghana

## Abstract

It is estimated that about 50% of people in low- and middle- income countries who require rehabilitation do not get it. Multidisciplinary rehabilitation services led by Physical and Rehabilitation Medicine (PRM) physicians have been shown to improve functioning, independence and the quality of life of persons with reduced functioning or disability. However, there is a dearth of PRM physicians in low to middle income countries (LMICs), particularly in sub-Saharan Africa. One potential solution to this lack of specialists is the establishment of PRM training programs, which are currently lacking. The International Rehabilitation Forum (IRF) developed and implemented a fellowship program to train physicians in rehabilitation medicine and has been successful in Ghana, Ethiopia and Cameroon, all LMICs in sub-Saharan Africa. However, ongoing challenges include inadequate PRM trainers, availability of logistics and services for hands on experience, and funding. The fellowship program has a promising future and an ultimate goal of having locally trained fellows leading the program and expanding it to other LMICs. There has however been no publication of the process followed to achieve this or of a similar process undertaken anywhere in Africa. The process followed in this publication highlights the journey from engaging stakeholders to the admission of new and current fellows in training.

## Introduction

There is an increasing unmet need for rehabilitation globally. The WHO estimates that about 50% of people do not receive the rehabilitation they require (1). This need is particularly high in low- and middle- income countries (LMICs) including those in sub-Saharan Africa. The reasons for a high percentage of unmet rehabilitation needs are multifactorial. One reason is rising non-communicable diseases in LMICs which are often associated with complications leading to disabilities (2). This is partly due to the negative culture of poor health screening. The quality of healthcare is also improving in these countries so the ageing population is increasing with its resultant reduction in functioning (3). Lastly, a high rate of road traffic accidents partly from poorly constructed roads is resulting in disabilities (4).

One strategy for addressing rehabilitation needs in high-income countries is for rehabilitation to be provided by a multidisciplinary team of rehabilitation professionals including occupational, physical, speech and language therapists, orthotists and prosthetists as well as rehabilitation nurses all led by a PRM physician. This approach to care has been shown to improve functioning, independence and the quality of life of persons with disabilities or reduced functioning (5). With the exception of the northern African countries of Morocco, Tunisia and Algeria, there is a dearth of PRM physicians in many countries in Africa including sub-Saharan Africa where the few PRM physicians are found in French speaking countries and mostly have partnerships with France for their training programs (6).

The establishment of PRM training programs in sub-Saharan Africa plays a very important role in increasing the number of PRM physicians and thus improving access to care. However, PRM training programs are lacking in a huge part of sub-Saharan Africa. A study published by Haig et al in 2009 revealed that there were only six PRM physicians in the whole of sub-Sharan Africa and this did not include Ghana (7).

The lack of PRM physicians meant that persons with reduced functioning missed out on comprehensive and coordinated rehabilitation in majority of sub-Saharan African countries where disability is stigmatized. This resulted in persons with disabilities being abandoned by family or ending up on the streets begging for alms to survive.

This paper chronicles the development of PRM as an important and vital specialty in sub-Saharan Africa using Ghana as a case study. The paper highlights the successes and challenges to improving access to PRM care through the establishment of a training program while providing recommendations for building on this approach.

## Method

### Bridging the gap

In response to the shortage of PRM providers and lack of medical rehabilitative care in LMICs including those in sub-Saharan Africa, the International Rehabilitation Forum (IRF), a non-for-profit organization, was created in 2009 by American and international rehabilitation advocates. The purpose of IRF is to build relevant rehabilitation medicine practices in low-resource and isolated regions.

The IRF utilized grass roots efforts and disruptive innovation as a means to challenge the status quo of rehabilitation in low resource settings. Examples of these efforts include identifying and mentoring rising leaders in LMICs, researching and publishing on disability policy, creating and distributing free videos on disability, and hosting “world congresses” in LMICs to foster local partnership (8). More recently, though COVID-19 largely prevented in person gatherings, the IRF held cost-free virtual global conferences during the pandemic. Over 60 PRM professionals from 25 countries joined the virtual conferences to discuss building sustainable rehabilitation programs in low resource settings, promoting physiatry to medical school students, and creating global rehabilitation curriculums. While many ministries of health in LMICs recognize a need for improved rehabilitation, there is often a lack in domestic resources needed to build programs (9–11).

To help address resource needs in sub-Saharan Africa, the IRF developed a plan to for a PRM fellowship training program utilizing international resources from high-income countries. In this case, the predominant resource utilized was teaching personnel for education. International educators dedicated their time to train interested physicians in sub-Saharan countries to become PRM experts and help construct sustainable programs. This model of international partnership between high-income and LMICs has been described by other medical specialties as well (12, 13).

The development of a fellowship program was based on a decade of exploration and trials. First explorations revealed a shocking lack of PRM specialists, formalized in an article that found only six specialists in all of sub-Saharan Africa. This article, which exposed failures of health systems, governments, non-government organizations and the WHO; was published simultaneously by five international journals as a call for action (7, 14–17).

To develop a strong and sustainable program it was important for the team to understand the socio-political environment, build more of a case for change, and get approval and support. To achieve this, African ex-patriates, including the Ghana Medical Rehabilitation Group, met monthly with IRF leaders, orienting them to culture, politics, and opportunities. There was initial fundraising to begin the program. African-born American-trained PRM specialists were identified and recruited as liaisons. Research publications co-written by African and American PRM physicians demonstrated the underrepresentation of disability in government epidemiological data (18), pointed out the inadequacy of trauma (7) and cancer (19) rehabilitation, explored Africa's need to build rehabilitation (20), and made the case for economic benefit to hospitals (21). Numerous trips identified rehabilitation supporters including neurologists, neurosurgeons, pediatricians, clinical directors of health and ministers of health in ministries and health institutions in countries. An important universal barrier was that in each country the premier government hospital was not capable or supportive of this change in healthcare.

The program only moved forward when African physicians with a passion were made aware of the IRF. A team of physicians including an Ethiopian doctor as well as the then Ethiopian minster of health and now WHO secretary general visited the United States. During that visit they met with IRF leadership and encouraged development of an on-line fellowship with a promise to approve it through the ministry of health. In Ghana, a dedicated physician associated with the IRF identified PRM could be moved forward as a 2-year subspecialty if developed as a “Sports, Exercise and Rehabilitation Medicine” program. Both countries had a very stringent, but somewhat different criteria for specialty training and a program was designed to fit both systems.

### Curriculum

Currently, little is known about the best curriculum to teach PRM on a global scale. A literature search for a structured curriculum for PRM training globally or *via* distance learning yielded no results. The initial IRF fellowship program was loosely organized around the Accreditation Council for Graduate Medical Education (ACGME) core competencies and modeled after residency training programs in the United States, headquarters of the IRF. The curriculum included components of the International Society of Physical and Rehabilitation Medicine's (ISPRM) core curriculum and competencies published in 2019 (22) with adaptations to also provide PRM training on conditions common in the African setting such as clubfoot and tuberculosis of the spine. The IRF also utilized the International Classification of Functioning, Disabilities and Health (ICF) as the basic tenet underlying the training of fellows (23). This was to teach fellows to approach rehabilitation from a biopsychosocial point of view, linking a person's level of functioning to the strong interaction between their health condition, environmental factors, and personal factors. One long term goal was to not only train these physicians as experts in PRM, but also as locally trained educators to form a core teaching staff in country to sustain the program long term.

**Figure 1:**
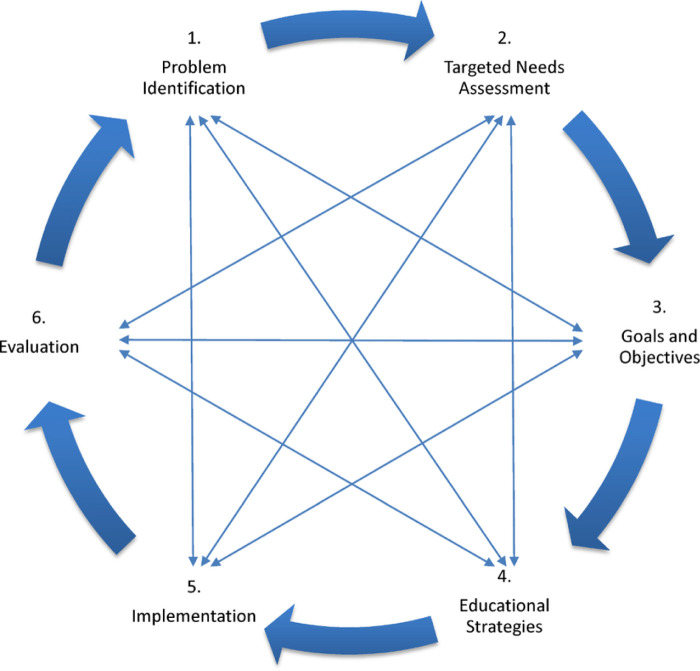
KERN cycle for medical curriculum development (24).

To develop a robust educational curriculum the IRF leadership recruited a fellowship director and a co-director. The framework of Kern's six step approach to curriculum development was then employed (Figure 1). (1) Problem identification and general needs assessment; (2) Targets needs assessment; (3) Goals and objectives; (4) Educational Strategies; (5) Implementation; and (6) Evaluation and feedback (24). Realizing that curriculum development is a dynamic process, leadership brainstormed ideas in a non-linear process, allowing thoughts and ideas to progress being ever mindful of three things: First, that all the fellows are already expert internal medicine/family physicians in their home countries and are dedicated to building on their current knowledge base to learn the specialty of PRM. Second, that in country populations and medical conditions although similar, may be different than those in the US and that resources such as equipment, access to care, medications, financial access to services and cultural norms and attitudes will influence the educational needs of trainees differently in different countries. Third, that core clinical training taking place in country will focus in part on orthopedic, sports medicine, neurologic conditions and orthotics and prosthetics and as such the goal of the didactic sessions is to instill knowledge and core thought processes based on PRM principles, establishing a “Rehabilitation mindset”.

**Figure 2:**
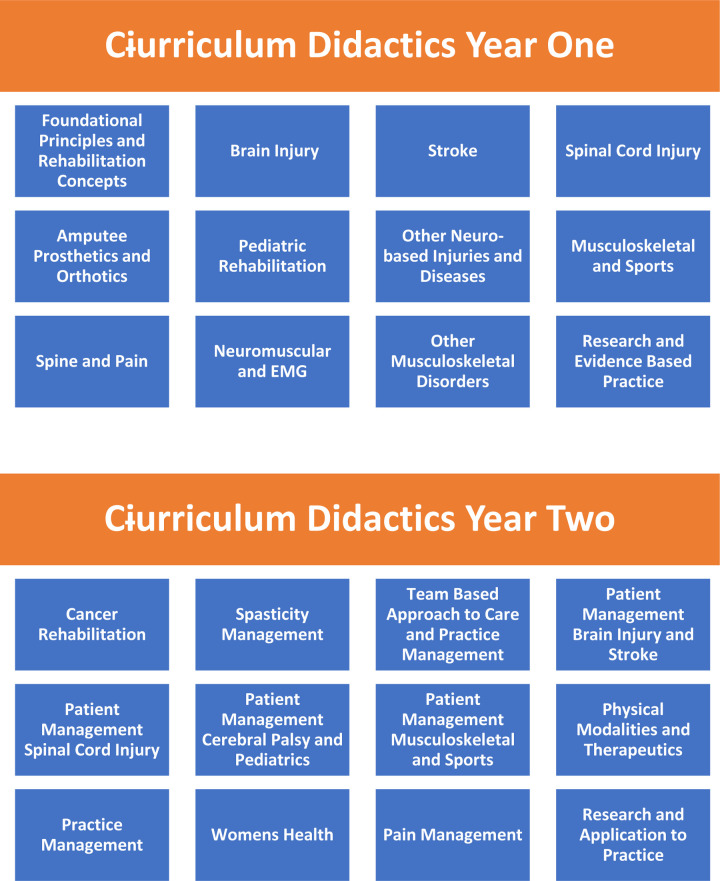
Summary of curriculum didactics.

The curriculum content for year one focused on covering core subjects with year two taking a deeper dive into each subject with more focus on case-based learning and fellows own case discussions (Figure 2).

Once a core subject matter and principles outline was developed, the training team recruited content experts for each subject globally. The methodology for teaching included a multimedia approach. Content expert power point and video-based lectures were pre-recorded and accessible online to allow flexible access to learning modules. The online formats were standardized to a universally downloadable and viewable format to accommodate operability on multiple device platforms with consideration of potential connectivity issues. One hour live (*via* internet) weekly didactic sessions with experts reviewing core content from the lectures and facilitating discussions of application to practice occurred. Homework assignments to reinforce learning concepts or to promote exposure to rehab mindset conceptualizations were made. Additionally, an on-line discussion forum was established to bring cases and questions to both fellow learners and content experts and to share clinical scenarios. A bank of core articles and assessment scales was also made accessible *via* the internet for all learners. Pre and post-tests were performed to assess acquisition of new knowledge. The in country sponsoring institutions were ultimately responsible for clinical experiences for the learners, but specific recommendations were made by leadership for experiences the fellows should seek out such as joint injections or exposure to EMGs. In the last 6 months of training, fellows completed their dissertation research and graduated or sat for their board exams depending on their medical college's guidelines.

The development of the curriculum for the fellowship program was also structured around equipping fellows with the skills required to prepare and present on common PRM topics both to colleagues and at conferences as well. Fellows also developed skills which enabled them to teach basic PRM to medical students and allied health professionals. This skill was vital in increasing medical students' exposure to and knowledge about PRM as a medical specialty which previously was missing.

## Results

### Successes and challenges

As of this publication, the fellowship has graduated four fellows, one from Ghana and three from Ethiopia. The current fellowship includes two cohorts with cohort one including three fellows (Ghana, Ethiopia and Cameroon) and cohort two including three fellows, all from Ghana. The political process involved in the establishment of a PRM fellowship in other African countries has been challenging. Cameroon has yet to formalize the training, and as such their fellow will receive an IRF diploma without board certification of the specialty of PRM. South African colleagues intend to join the next cohort of trainees despite no formal structure under the MOH and will work to gain government recognition only after a number of graduates can advocate for their careers.

As a requirement of developing the educational program it was requested that host hospitals commit their trainees' time, have allied health and specialist physician buy-in, and establish a small inpatient rehabilitation unit. Despite promising initial conversations neither of the SSA host hospitals have made gains in establishing space for inpatient rehabilitation at this time. The opportunity to create rehabilitation wards is immediate with post operative and neurologically injured patients having long stays and future rehabilitation units providing a value-added service freeing surgical specialists and internal medicine beds and time to focus on acute needs. Without inpatient rehabilitation wards for hands on patient care experiences it is difficult to adequately train the fellows and development of functioning rehabilitation teams will be a daunting task as the fellows work to establish PRM as a specialty in their country. Once there is a core PRM trained group of providers, nurses and therapists trained in rehabilitation concepts and techniques will be necessary to appropriately treat injured and disabled patients for optimal outcomes.

Fellows have taken on the mantle of academic and political leadership in African rehabilitation. As a group they have published a number of papers on rehabilitation in Africa (6, 25, 26). They also mobilized at the beginning of the COVID-19 pandemic to put out the very first international rehabilitation triage tools (27). Ghana's first graduate has taken important roles in the WHO, the ISPRM, and the IRF along with the role of fellowship director in Ghana. One of Ethiopia's first graduates is successfully developing an inpatient rehabilitation program and Cameroons soon to graduate fellow has formed a Cameroon rehabilitation consortium including research and education missions, while others are influencing policy through their current roles, including a family practice residency director and a leprosy hospital medical director. These very first pioneers had passion and career-paths in mind. Each felt a need for credibility. Clinical career paths going forward appear to tie extensively to prior passion. They include neurorehabilitation, orthopedic inpatient rehabilitation, cancer rehabilitation, leprosy, pediatric rehabilitation and sports rehabilitation.

Challenges have been faced in the program running and development, with a primary obstacle being lack of adequate in country expertise in PRM and as such awareness of the field. One goal of the program is for graduates to not only practice PRM, but to also become the mentors and clinical teachers for future fellows. Until that occurs, training will continue to be led by those practicing outside the host countries.

Although an initial hope was for the program to largely be taught in country by African orthopedic surgeons, neurologists, and physiotherapists, none of these specialists had ever interacted with a doctor specializing in functioning (PRM) so the current distant-led program was chosen. Bridging this medical cultural gap is a major focus of the first 8 weeks of the fellowship. Questions of “how does a PRM consultation add to good care by a neurologist or orthopedic surgeon?” are answered with basic competency training in bowel, bladder, swallowing, nutrition, skin, and mental health. Introductions to how a PRM team functions including introduction to allied health professions, some of whom do not exist in many SSA countries is provided as is the multidisciplinary team approach to care which is a basis of PRM. Training fellows in the basic science of exercise, functioning, and outcomes is paramount to the rehabilitation mindset and without this orientation fellows risk returning to a “diagnose-treat-cure” process rather than also including functioning and quality of life in their treatment plan.

Because many allied health specialties are not universally present in SSA and others training may vary widely, education regarding the potential of these roles at times was theoretical. Providing an introduction to what different specialties, such as speech therapy, may contribute to treatment of a stroke or brain injury patient expanded long-term thinking and planning for the fellows as they develop not only their own practice but are poised to lead the profession of PRM in their own countries. The cultural aspect of rehabilitation, advanced in more mature rehabilitation systems by other team members such as nurses, recreational therapists, occupational therapists or rehabilitation counselors, appeared as new and exciting visions for health care, for the fellows, who are uniformly passionate about disability rights.

This gap between established rehabilitation and the African reality leads to a critical need for the fellows to have opportunities to learn hands on in functioning rehabilitation units and teams in observation overseas. Initial fellows who had this opportunity came home confidently advocating for sophisticated rehabilitation, having seen PRM in a mature light. More technical or hands on aspects of learning are difficult to teach without travel by either the trainee or the trainer. These include spinal injections, electrodiagnosis, neurolysis, serial casting, manual exam and other skills. The variable availability of equipment such as ultrasound machines, EMGs, fluoroscopy, etc. poses a challenge for trainees to learn interventions and diagnostic techniques core to the profession internationally. Funding travel was and is a very large barrier. Additionally, as the first in their nations, current fellows have yet to meet and work with another specialist in the field of PRM.

## Ghana as a case study

Ghana, a country in West Africa was for many years without a PRM training program until the year 2018 when the first such program was established. Prior to this, there was no PRM physician in Ghana. Christian et al did a study in 2016 focusing on rehabilitation in Ghana and noted that only 17% of the patients who required rehabilitation in Ghana's second biggest teaching hospital and referral center received it. And this care was in the form of only physical therapy provided once a day for less than a week (28).

In 2018, the Ghana College of Physicians and Surgeons (GCPS) through its Family Medicine faculty partnered with the IRF and approved a “Sports, Exercise and Rehabilitation Medicine” (SERM) fellowship program to train Family Physicians in the field of PRM and Sports Medicine. The 2-year training in SERM in Ghana was adequate to train physicians because unlike other countries where the training takes 4 years with trainees coming straight out of medical school, the physicians being trained in the IRF program had already undergone a 3-year training in Family Medicine, qualifying as specialists and had competencies in patient safety and quality patient care, interpersonal skills and communication, medical ethics and public health, quality assurance, professionalism and research skills among others, similar to what has been outlined in the ISPRM's PRM core competency document.

The program utilizes collaboration between the GCPS, IRF physician members and IRF's recruited experts in the discipline of PRM around the country to facilitate and build faculty training and create local training programs led by alumni. In addition to the online lectures, fellows training in the program go through clinical rotations in other medical specialties including Neurology, Neurosurgery, Trauma and Orthopedics, Radiology, Rheumatology, Cardiology and Psychology. They also spend time in the largest prosthetics and orthotics center in West Africa learning about the common injuries presented as well as the various products provided. Prior to COVID-19, educational exchange visits between Ghanaian physicians and academic centers in the United States (University of Michigan) was a part of the fellowship training. However, in lieu of travel restrictions brought about by the COVID-19 pandemic, the fellowship focused solely on virtual educational exchanges.

The delivery of PRM training through the Sports, Exercise and Rehabilitation Medicine (SERM) sub-specialty program under the GCPS in Ghana presented the opportunity to incorporate PRM into the training of medical students. This approach in addition to exposing the students to this specialty field, also guides them towards pursuing a career in PRM.

Again, with the WHO and countries pushing for Universal Health Coverage (UHC) to reach lower income populations with subsidized healthcare, it becomes imperative that medical doctors often encouraged by medical schools to work largely at the primary care level are given some pre-service exposure to PRM in line with the framework of action for UHC in Africa. The GCPS' faculty of family medicine has an existing family medicine immersion program available within the University of Ghana Medical School (UGMS), Ghana's oldest medical school. With PRM training in the GCPS being under the faculty of family medicine, this turned into a fortuitous opportunity to introduce PRM to senior clerks at the L600 level in 2021. Within a 1-week senior clerkship program, one of the morning tutorial sessions is devoted to SERM, during which a fellow introduces the fundamental concepts of PRM laced with some problem-based scenario learning. In 2021, about 200 L600 students at UGMS received this immersion in SERM, in four divided cohorts. Just within the same year, one of the private medical schools also in Accra has incorporated a similar program into its undergraduate medical program, with their smaller number of final year students being trained in a single cohort.

The PRM training built within the SERM program however has not been without challenges. The first fellow faced the pioneering challenge of promoting PRM among colleague physicians. Establishing a practice and developing a clinic and inpatient unit to treat patients is paramount not only to the future outcomes for patients but also to improve hands on and case-based learning opportunities for the training program. To improve visibility both oral and poster presentations highlighting PRM and its role in managing various conditions (Cerebral Palsy, Stroke, Spinal Cord Injuries, Back Pain) were made at GCPS conferences and continuous professional development workshops. Presentations were also made at the clinical meetings of the various PRM associated specialties including orthopedics, paediatrics and internal medicine. Teaching sessions were organized for family physicians who were the specialists with the largest encounter with persons who would require rehabilitation. These activities have resulted in basic PRM training also being incorporated into the 3-year Family Medicine residency training involving lectures and clinical rotations in the fellow's practice thus further exposing them to the field of PRM.

## Discussion

The long-term goal of the IRF is to help develop local Ghanaian and other LMICs fellowship and training programs for PRM by training and supporting rehabilitation specialists. IRF's main purpose will become that of a supporting partnership to local training programs, though there will be continued educational exchanges for the benefit of both LMICs and high-income countries.

The approach of incorporating PRM training within an already established undergraduate program for family medicine, looks to be a viable model, although it is still too early to assess the full impact, and promises to yield huge dividend in the near rather than long-term future. It also bodes well for an LMIC like Ghana which needs to bridge the rehabilitation access gap very quickly within the context of UHC.

The IRF is currently working on the development of an online platform to house recorded didactics, journal articles, videos, and discussion platforms. Next steps in curriculum development include more direct assessment of the learner's knowledge, as well as program evaluation of the content and learning process. Further content banking of modules for enhanced access for repetition and reference. Additionally, increased collaboration with the sponsoring in-country institutions to better align didactic learning sessions, focused on building blocks of core PRM knowledge, with hands on clinical experiences. For example, aligning stroke and brain injury didactics with neurology rotations.

The ultimate goal of the IRF fellowship is not to train African fellows. It is to train Africans who can train the first generation of African PRM physicians. The early success bodes well for this vision, as graduates have already become active leaders and teachers.

## Data Availability

The original contributions presented in the study are included in the article/Supplementary Material, further inquiries can be directed to the corresponding author.
